# Innovations in Inventory Management to Improve the Profitability of Local SMEs

**DOI:** 10.12688/f1000research.168900.1

**Published:** 2025-09-02

**Authors:** Víctor Hugo Puican Rodríguez, RITA DE JESUS TORO LÓPEZ, Waldemar Ramón García Vera

**Affiliations:** 1 Universidad Cesar Vallejo, Trujillo, La Libertad, Peru; 2 Universidad Cesar Vallejo, Trujillo, La Libertad, Peru; 3 Universidad Cesar Vallejo, Trujillo, La Libertad, Peru

**Keywords:** Inventory, profitability, control, measurement, financial efficiency.

## Abstract

**Background:**

Efficient inventory management is critical for the financial and operational sustainability of micro and small enterprises (MSEs) in Peru, particularly in the wake of the COVID-19 crisis. In Bagua, many of these businesses lack digital systems for real-time tracking, impairing decision-making and reducing profitability. The absence of clear safety-stock policies and strategic replenishment guidelines often leads to emergency purchases at high prices and imbalances between excess and shortage of goods.

**Method:**

This study employed a quantitative, descriptive-explanatory design. A convenience sample of 83 MSEs in Bagua yielded 200 valid responses from key participants (managers, warehouse staff, accountants, and assistants). A 21-item Likert questionnaire, validated by experts, captured perceptions of inventory control, valuation methods, record-keeping, and measurement. Data were analysed in SPSS v27 using descriptive statistics and multiple linear regression to assess the impact of these inventory practices on profitability indicators (ROA, gross margin, and ROE).

**Results:**

Descriptive analysis revealed moderate performance across all four inventory dimensions, with mean scores ranging from 2.37 to 2.62. The regression model explained 40.2 % of the variance in profitability (R ^2^ = 0.402; p < 0.001). Inventory measurement emerged as the strongest predictor (β = 0.383; p = 0.001), followed by inventory control (β = 0.257; p = 0.013).

**Conclusion:**

Neither valuation methods nor record-keeping showed statistically significant effects. Accurate inventory measurement and control are key drivers of profitability for Bagua’s MSEs. Adoption of real-time tracking technologies and revision of valuation and recording practices are recommended to enhance financial performance. Given the study’s cross-sectional design and geographic focus, future research should employ longitudinal approaches and comparative analyses across different regions and sectors.

## Introduction

Efficient inventory management has become an essential practice for ensuring financial and operational sustainability in micro and small enterprises (MSEs) in Peru, particularly after the crisis caused by the COVID-19 pandemic; the implementation of effective strategies for inventory control (IC) and measurement can generate tangible monetary benefits by reducing losses associated with expiration, deterioration, or theft of goods; however, a significant number of these companies lack digitalized systems that allow real-time inventory tracking, directly affecting decision-making and reducing business profitability (P) ( Vahdani & Sazvar, 2022; Wellner & Lakotta, 2020); MSEs need to be flexible with their inventory management because demand changes quickly and is sensitive to price; however, many of them have trouble because their managers aren’t very experienced and their shareholders aren’t very committed; this situation hurts key performance indicators like Return on Assets (ROA) and Return on Equity (ROE) ( Li & Mizuno, 2022); furthermore, the lack of safety stock policies leads to rushed acquisitions at high prices, increasing the final product cost and significantly reducing sales ( Johnston et al., 2022; Rueda et al., 2022; Singh et al., 2022).

In Bagua, a city representative of the reality faced by many Peruvian MSEs, inventory management encounters particular challenges, such as product shortages or excesses; companies use tools like the “Q-mode” method, which constantly monitors inventory levels, although it does not provide clear guidelines on the optimal quantity and timing for restocking goods, requiring more strategic relationships with suppliers ( Carazas et al., 2019); additionally, deficiencies in planning hinder the ability to effectively forecast demand, directly impacting operational expenses, delivery times, and perceived quality key factors determining business profitability ( Jurado et al., 2021; Wilson et al., 2022; Wilson, 2023).

This research aims to determine the impact that inventory control and measurement have on the profitability of micro, small, and medium-sized enterprises (MSEs) in Bagua, Peru; the primary objective of this study is to determine how various methods of valuing inventory (including first-in, first-out (FIFO), last-in, first-out (LIFO), and average cost), as well as maintaining accurate records of inventory and maintaining strict control, have an effect on important measures of profitability such as return on assets (ROA), gross margin (GM), and return on equity (ROE).

The objective was to explore the impact of inventory control, inventory valuation methods, inventory control recording, and inventory measurement on the profitability of MSEs in Bagua, Peru. To describe the multidimensional statistical characterization of internal control and its impact on key profitability indicators of MSEs in Bagua, Peru.

The most important thing this paper does is show how inventory management directly affects a business’s ability to make money in a certain area and type of business; it also adds to the body of knowledge about how to run a small business’s finances and operations better, and it gives them suggestions on how to make their results much better.

### Literature review

Several studies have highlighted the significance of effective inventory management in significantly increasing company profitability, especially for SMEs; effective inventory management significantly affects financial performance, operational profit margin, and net income in various production sectors; the key is to maintain an appropriate inventory level to prevent shortages and overstocking ( Ahmad et al., 2022; Anisere & Bodunde, 2021; Sultan, 2021; Gamariel & Annet, 2021; Golas, 2020).

Mathematically simple models such as the Economic Order Quantity (EOQ) are frequently employed to determine optimal stock levels and reduce operational costs associated with purchasing, warehousing, and stockouts ( Çalışkan, 2021; Condeixa et al., 2020; Nobil et al., 2023; Ayatollahi & Jafari, 2022; Liao et al., 2019); on the other hand, the Wilson model takes into account volume discounts and variable storage costs, adding a temporal dimension, which makes it more flexible in response to changing and unpredictable demand environments ( Samadi et al., 2020; Kazemi et al., 2018; Wray et al., 2023; Shepherd et al., 2023; Liu et al., 2020).

Despite queuing theory’s original intent, researchers have found ways to apply it to inventory management. This has led to improvements in replenishment times and shorter lead times ( Ahmed et al., 2019; Wu et al., 2023; Petrovic et al., 2023; Ala et al., 2023; Qandeel et al., 2023); integrated inventory management, which includes accurate valuation methods, comprehensive controls, and regular measurements using key performance indicators (KPIs), improves operational visibility, optimizes space utilization, and reduces costs ( Chunxia & Shunfu, 2018; Chen & Kong, 2019); finally, from a business lifecycle and Porter’s five competitive forces point of view, MSEs can clearly see their competitive landscape and change their inventory strategies to make the most money, which gives them advantages like being different and running their businesses more efficiently ( Pizzan et al., 2022; Jerónimo et al., 2022; Nirmala et al., 2022; Becerra et al., 2022); similarly, the sustainable growth model provides a framework for balancing growth and profitability, making decisions that ensure long-term financial stability ( Epizitone & Nxumalo, 2021; Drakaki & Tzionas, 2021; Nozari et al., 2022; Gammelli et al., 2022; Preil & Krapp, 2022; Walt & Bean, 2022; Moncayo & Tarrillo, 2020; Zhou, 2023; Jiang et al., 2022; Khuong & Anh, 2023; Dias et al., 2023; Baxter, 2019; Maartens & Hutmacher, 2022; Fernandez et al., 2020; Chien-Chiang et al., 2023; Henderson & Loreau, 2023; Hu & Liu, 2022; Harbiankova & Scherbina, 2021; Lan et al., 2019; Copeland & Weston, 1988; Pissan et al., 2022; Luna et al., 2021; Mundt et al., 2022; Merino et al., 2021; Setianto et al., 2022; Belloso et al., 2021; Caiza et al., 2020; Moncayo & Tarrillo, 2020; Ramón & Bañón, 2022; Sánchez et al., 2022; Kogan et al., 2022; Flores, 2019).

## Methods

### Focus, design and type of research

This research follows a quantitative approach, complemented by a descriptive-explanatory design, directly aligned with the established objectives; the use of this approach allowed for the systematization and statistical analysis of the data, facilitating the generation of generalizable and verifiable results through hypothesis testing; additionally, the study did not limit itself to merely describing phenomena but also measured and analysed the causal relationships between the different variables and dimensions involved; as an applied study, the results provided direct practical implications, offering valuable information for more informed decision-making regarding inventory management and its impact on profitability.

### Study population and sample

A total of 83 MSEs in the city of Bagua were contacted, resulting in the inclusion of 415 individuals, as five key members from each MSE were considered: the manager, warehouse staff, general accountant, and accounting assistants; consent from the managers was sought for participation in the questionnaires designed for the research; these companies represented various sectors, including hardware stores, multipurpose services, and construction companies; using the finite proportion formula, the final sample consisted of 200 individuals, representing 40 microenterprises, with participants holding roles such as managers, warehouse supervisors, accountants, and accounting assistants.

### Sampling, inclusion and exclusion criteria for the study

A non-probability convenience sampling method was employed, selecting the most accessible MSEs for the researchers; inclusion criteria for selecting MSEs included: voluntary participation, registration as active businesses, up-to-date payments in accordance with the Unique The study required MSEs to be registered with the Taxpayer Registry and to have a minimum of five active employees; the study excluded MSEs that failed to meet these criteria or couldn’t commit the required time.

### Data collection technique and instrument

Prior to the final application of the questionnaire, a pilot test was conducted with a reduced sample to evaluate the reliability of the items using Cronbach’s alpha coefficient; this test allowed for the adjustment and refinement of the questionnaire items to ensure they were clear and consistent, ensuring the quality and reliability of the data collected; the second section of the questionnaire explained the scales used for data collection, such as the Likert scale, which offered five response options: “Never” (scored as 1), “Rarely” (2), “Sometimes” (3), “Almost always” (4), and “Always” (5); the third section measured participants’ perceptions and attitudes regarding the central theme of the study through Likert scales and multiple-choice questions; finally, the fourth section included items aimed at gathering information about participants’ experiences and behaviour’s related to the investigated phenomenon.

### Validation of the instrument and statistical methods used

The questionnaire, consisting of 21 items, was previously validated by three experts holding master’s and doctoral degrees; after validation, the collected data were organized in an Excel spreadsheet and transferred to SPSS 27 for statistical analysis; the first two specific objectives were addressed using descriptive statistical methods to examine the levels of the respective variables and dimensions; subsequently, inferential statistical analysis was applied to determine whether the data were parametric or non-parametric, which was crucial for hypothesis testing and analysing the relationships between variables.

## Results and discussion

### Results

Table 1 shows a summary of the different types of statistics that describe inventory management and how it directly affects key indicators of MSEs’ profitability in Bagua; a moderate performance is observed across all evaluated dimensions, with inventory measurement showing the highest mean value (2.62); this finding suggests that inventory measurement is prioritized by firms in their operational practices; the negative skewness across variables indicates a trend toward higher self-assessed performance ratings, especially in inventory measurement; in important areas related to profitability, the mean scores for all variables fall between 2.37 and 2.62, indicating generally moderate performance; additionally, the median value of 3 further supports the assertion that the majority of firms achieve acceptable performance levels; the negative skewness values, which are most noticeable in Return on Assets (ROA: -0.947) and Gross Margin (-1.007), suggest that while many firms report pretty good performance, a smaller group with worse performance may be changing the overall mean.

**Table 1.** Multidimensional statistical characterization of inventory management and profitability indicators.

Variables		IC	IVM	ICR	IM	P	ROA	GM	ROE
N	Valid	200	200	200	200	200	200	200	
	Lost	0	0	0	0	0	0	0	
Mean		2,37	2,40	2,50	2,62	2,44	2,49	2,55	2,54
Median		2,00	2,50	3,00	3,00	2,50	3,00	3,00	3,00
Mode		2	3	3	3	3	3	3	3
Standard deviation		,619	,672	,665	,647	,599	,665	,608	,625
Variance		,384	,451	,442	,419	,359	,442	,369	,390
Skewness		-,434	-,665	-,965	-1,448	-,565	-,947	-1,007	-1,027
Standard		,172	,172	,172	,172	,172	,172	,172	,172
Kurtosis		-,650	-,633	-,228	,833	-,594	-,257	,003	,001
Standard error of Skewness		,342	,342	,342	,342	,342	,342	,342	,342
Rango		2	2	2	2	2	2	2	2
Minimum		1	1	1	1	1	1	1	1
Maximum		3	3	3	3	3	3	3	3

Note: Data obtained from SPSS V27.

The variables in Table 2 related to inventory management explain about 39% of the differences in how profitable Bagua’s MSEs are; this is shown by an R-squared value of 0.402 and a significant correlation coefficient (R = 0.634, p < 0.001); inventory measurement emerges as the variable with the most substantial positive effect on profitability, displaying a standardized beta coefficient of 0.383 (p = 0.001); inventory control also significantly contributes to profitability, albeit to a lesser extent (Beta = 0.257, p = 0.013); conversely, inventory valuation methods and control records were statistically insignificant, suggesting limited direct influence on profitability within this context.

**Table 2.** Regression model summary, ANOVA, and standardized coefficients.

Variables	Summary of the model	ANOVA
R	0.634	
R ^2^	0.402	
Adjusted R ^2^	0.390	
Standard Error of Estimation	0.468	
Change in F	32.805	32.805
Sig Change in F	0.001	0.001
Durbin-Watson	1.525	
Regression		28.718
Residual		42.677
Coefficients	Standardised coefficients Beta	T/Sig.
IC	0.257	2.497/0.013
IVM	0.053	0.781/0.436
ICR	0.037	0.436/0.663
IM	0.383	5.036/0.001

Note: Data obtained from SPSS V27.

### Discussion

The study shows that residuals are close to a normal distribution, which proves a key regression assumption and shows that the regression model is robust and reliable; normality in residuals ensures the precision of estimates and strengthens the credibility of findings, particularly regarding inventory management practices and their significant relationship to profitability; the regression results underscore inventory measurement as the predominant factor influencing profitability, with a standardized coefficient Beta of 0.383; this aligns with extant literature highlighting effective inventory measurement as critical for profitability enhancement, notably within manufacturing and similar sectors Ahmad et al. (2022) and Gamariel and Annet (2021); additionally, inventory control also demonstrated significance (Beta = 0.257), corroborating findings from previous studies ( Sultan, 2021) the fact that inventory valuation methods and control records don’t have any statistical significance shows that these practices need to be carefully looked at again and maybe changed to fit Bagua’s business environment.

The empirical results underscore that inventory management variables, particularly inventory measurement, have a moderate yet meaningful impact on the profitability of Bagua’s MSEs; these findings confirm the critical role inventory measurement and control play in enhancing operational efficiency and financial performance. This research aligns with prior empirical investigations that report a positive association between efficient inventory management and enhanced profitability Nobil et al. (2023) and Wray et al. (2023); such alignment reinforces the notion that improved inventory practices consistently yield measurable financial benefits across diverse economic sectors and regions.

Implementing precise inventory measurement practices could significantly enhance resource optimization and profitability within Bagua’s MSEs; firms are encouraged to integrate advanced inventory tracking technologies to streamline processes, reduce operational expenses, and improve competitive positioning; additionally, revisiting current inventory valuation methods may further improve accuracy and profitability outcomes.

This research is constrained by its cross-sectional design and geographically focused sample, potentially limiting the generalizability of findings; furthermore, the static nature of cross-sectional analysis precludes exploration of longitudinal causal relationships and profitability trends over time.

Future research should adopt longitudinal designs to investigate the sustained effects of inventory management practices on MSE profitability over extended periods; comparative analyses across different regions or industrial sectors could further enrich understanding and yield more profound insights into best practices and their adaptability within varied business environments.

## Conclusions

This study confirms that efficient inventory management, particularly in the areas of measurement and control, is a key factor in enhancing the profitability of MSEs in Bagua; by implementing a more precise inventory control system, companies can optimize their processes, reduce costs, and improve resource allocation, leading to greater organizational and financial efficiency; the results obtained align with previous research, which also emphasizes the importance of accurate inventory measurement as a driver of profitability for small and medium-sized enterprises; however, significant gaps remain, particularly in the areas of inventory valuation methods and record-keeping, which require specific adjustments and adaptations to maximize their impact; addressing these challenges through strategic investments in staff training, the implementation of advanced technologies, and improvements in inventory management practices will enable MSEs to optimize their performance and compete more effectively in the market; in this way, businesses in Bagua and similar regions can accelerate their transition to more efficient, profitable, and competitive business management.

## Ethics and consent

This research was approved by the Ethics Committee of César Vallejo University (Resolution No. 447-2023-VI-UCV of the Vice-Rectorate for Research), in accordance with the principles of the Declaration of Helsinki. All procedures complied with national and international ethical standards applicable to research involving human participants.

Written informed consent was obtained from all participants before administering the questionnaire. All respondents were over 18 years of age and were fully informed about the objectives of the study, the voluntary nature of their participation, the confidentiality of their data, and the exclusive use of the information for academic and scientific purposes.

## Data Availability

All data supporting the results of this study including the values underlying the reported means, standard deviations, and other measures; the values used to generate the figures; and the data points extracted from images for analysis are available in the Data availability the Victor Puican repository. **Process identifier** https://doi.org/10.23728/B2SHARE.C7BA1BDAF7B8423A8D0E467A446EB0F9 ( Rodriguez et al., 2025). **Additional materials and data** The additional data used in this study, including the complete questionnaire, instrument application guide, and supplementary tables of statistical results, are publicly available in the same data repository. **Process identifier** https://doi.org/10.23728/B2SHARE.C7BA1BDAF7B8423A8D0E467A446EB0F9 ( Rodriguez et al., 2025). These materials allow for open replication and review of the study. The full dataset is accessible without restrictions or embargoes under a CC-BY 4.0 license.
