# Innovations in Inventory Management to Improve the Profitability of Local SMEs

**DOI:** 10.12688/f1000research.168900.2

**Published:** 2025-11-07

**Authors:** Víctor Hugo Puican Rodríguez, RITA DE JESUS TORO LÓPEZ, Waldemar Ramón García Vera

**Affiliations:** 1 Universidad Cesar Vallejo, Trujillo, La Libertad, Peru; 2 Universidad Cesar Vallejo, Trujillo, La Libertad, Peru; 3 Universidad Cesar Vallejo, Trujillo, La Libertad, Peru

**Keywords:** Inventory, profitability, control, measurement, financial efficiency.

## Abstract

**Background:**

Efficient inventory management is a critical internal capability for ensuring the financial sustainability of micro and small enterprises, especially in emerging economies affected by post-pandemic disruptions. In Bagua, Peru, MSMEs often lack digitised control systems, leading to frequent stock imbalances, rushed purchases at high prices, and lower profitability; a deeper understanding of inventory practices that drive financial performance can contribute to more informed and strategic decision-making.

**Method:**

This quantitative, descriptive, and explanatory study examined the effects of four components of inventory management—control, valuation methods, control records, and measurement—on profitability; a convenience sample of 83 MSMEs yielded 200 valid responses from key personnel involved in inventory decisions; A 21-item Likert scale validated by experts was applied, and the data were analysed using descriptive statistics and multiple linear regression in SPSS v27. Profitability indicators included return on assets (ROA), gross margin (GM), and return on equity (ROE).

**Results:**

All dimensions of the inventory showed moderate levels of implementation (means: 2.37–2.62 on a five-point scale). The regression model demonstrated satisfactory predictive power (R ^2^ = 0.402; F = 32.81; p < 0.001). Inventory measurement was the strongest and only highly significant predictor of profitability (β = 0.383; p < 0.001), followed by inventory control, with a smaller but significant contribution (β = 0.257; p = 0.013). Inventory valuation methods and control records did not show statistically significant direct effects (p > 0.40).

**Conclusion:**

The findings highlight that decision-oriented measurement practices, supported by systematic control, are essential factors for the financial performance of micro and small enterprises; investment in digital monitoring tools, replenishment based on key performance indicators, and staff training could improve operational and financial efficiency; due to its cross-sectional design and localised context, future studies should incorporate longitudinal data and broader geographical comparisons to strengthen generalisation and explore possible mediation pathways between accounting-oriented practices and profitability.

## Introduction

Effective inventory management is a fundamental lever for working capital discipline and the profitability of MSMEs; beyond preventing shrinkage or obsolescence, inventory policies determine cash conversion and margin realisation through days of inventory outstanding (DIO), cash conversion cycle (CCC) and inventory profitability ratios such as GMROI; Recent reviews show that inventory practices, when complemented by technology and management expertise, substantially improve the operational performance of SMEs under conditions of resource scarcity ( Panigrahi RR, et al., 2024).

From a mechanistic point of view, reordering policies with continuous review (R, Q) and demand classification options are important for shortages, maintenance costs and service levels; in the case of intermittent or irregular demand, established evidence recommends Croston-type methods and their bias-corrected variants, which consistently outperform naive smoothing for low-turnover items common in local assortments ( Zhou, 2023; Wilson et al., 2022; Wilson, 2023).

### Research gap

Empirical associations between specific inventory practices and profitability have been documented mainly in large companies or urban settings; there is little evidence on micro and small enterprise (MSE) ecosystems in the Peruvian Amazon, where company size, informality, and logistical constraints may alter the magnitude of the effects (e.g., variable delivery times, higher risk of spoilage). In 2023, the Amazon region accounted for around 96% of micro-enterprises, with a predominance of commercial activities, conditions that amplify the operational impact of replenishment and record-keeping decisions ( Ministry of Production, 2024).

Why Bagua, Peru? Bagua is a representative province of the Amazon where the heterogeneity of the road surface and connectivity deficiencies complicate supplier delivery times and replenishment frequency, plausibly altering optimal targets (R, Q) and safety stock policies relative to coastal or metropolitan references. This context offers external validity for similar secondary cities, while providing policy-relevant insights ( Suárez et al., 2024).

### Contribution

The association between inventory control and measurement practices was quantified, emphasising accurate record keeping, safety stock policies and valuation methods consistent with IFRS/IAS 2 (i.e. FIFO and weighted average; LIFO is not permitted under IFRS) and profitability indicators (ROA, gross margin, ROE) in micro and small enterprises located in Bagua. By focusing on an underserved geographical area and aligning accounting treatment with IFRS, evidence was provided on how specific and applicable inventory control practices are linked to financial performance in constrained environments ( KPMG, 2021).

## Literature review

Inventory management is a fundamental operational capability within the resource-based view (RBV) framework: organisations reap financial benefits when internal resources, such as inventory control processes, ICT systems and forecasting skills, create cost advantages or improve responsiveness ( Barney, 1991; El Nemar et al., 2022). From a dynamic capabilities perspective, the ability to detect fluctuations in demand, take advantage of replenishment opportunities and reconfigure stock policies is particularly important for micro and small enterprises exposed to volatile environments, such as the Amazon region of Peru ( Teece, 2007; Chen et al., 2024).

### Definitions and mechanisms

Inventory control refers to coordinated decisions about the timing and quantity of replenishment, commonly represented by continuous review policies (R, Q), which determine stock-out costs, carrying costs, and service levels; basic profitability channels operate through days of inventory outstanding (DIO), cash conversion cycle (CCC), and inventory profitability ratios such as GMROI; safety stock policies mitigate delivery time uncertainty but must balance capital tie-up and service performance ( Kotecha and del Rio, 2025).

### Empirical evidence

Current studies have revealed a consistent positive relationship between CI practices and the profitability of micro and small enterprises, especially those where management skills and digital visibility remain scarce ( Li and Mizuno, 2022; Panigrahi A, et al., 2024). In terms of replenishment decisions, EOQ-type models and their extensions remain viable, low-cost solutions for companies with limited analytical capabilities, as they reduce ordering and storage costs ( Condeixa et al., 2020; Çalışkan, 2021). However, demand in small retailers is often intermittent or irregular, favouring Croston-type methods over naive smoothing to avoid systematic biases and service losses ( Syntetos et al., 2005; Erjiang et al., 2022).

Accurate record keeping and IFRS-aligned valuation methods (IAS 2), i.e. FIFO or weighted average, improve cost allocation, margin visibility and the quality of financial reporting ( Ala et al., 2023). Similarly, KPIs such as compliance rate, inventory turnover, and shrinkage rate have proven to be highly predictive of operating margins ( Sánchez-Córdova et al., 2024). Therefore, integrated inventory control systems, whether manual or digital, impact profitability not only through cost containment but also through strategic differentiation, especially in short-life-cycle categories susceptible to deterioration.

Despite these data, geographical coverage remains skewed towards the urban and well-digitised ecosystems of Asia and Europe, with limited evidence in secondary cities or Amazonian economies ( Granillo-Macías et al., 2024). The structural conditions of these environments—informality, long delivery times, concentration of suppliers—may moderate the link between inventory policies and profitability ( Singh et al., 2022). This contingency suggests that practices that are effective in metropolitan contexts may not produce similar effects in regions such as Bagua, Peru.

### Summary and gap

Previous studies corroborate that inventory capabilities create financial value, but few studies integrate RBV with dynamic capabilities to explain profitability outcomes in SMEs under the specific constraints of the Amazon; Consequently, this study examines how inventory control and measurement practices affect ROA, GM, and ROE in Bagua-based SMEs, addressing a geographical and theoretical gap with practical implications for similar contexts in developing economies.

## Methods

### Research design and rationale

A quantitative, descriptive and explanatory design was adopted, giving priority to inventory control ((R, Q), safety stock), valuation (FIFO or weighted average in line with IFRS/IAS 2), accuracy and timeliness in record keeping and inventory measurement (DIO, turnover, GMROI, compliance rate) as internal capabilities that link stock depletion, maintenance and replacement costs to profitability through the cash conversion cycle.

### Population and sampling frame

The target population consisted of micro and small enterprises registered and active in Bagua with ≥5 employees; 83 micro and small enterprises in the trade/services and construction sectors were contacted, inviting five informants per company (manager, warehouse manager, general accountant and two accounting assistants) to mitigate single source bias; inclusion required active tax registration, operational status, and willingness/commitment of time; the valid sample included 200 respondents from 40 micro-enterprises.

### Sampling strategy, limitations, and future research

Non-probability convenience sampling was used due to access restrictions and management authorisation; this implies coverage/selection bias and limits statistical generalisation; future studies should apply probability-based stratified sampling (by sector/size), multi-wave panels, and triangulate survey responses with accounting/ERP records.

### Variables and operationalisation

Inventory control comprised three dimensions: inventory valuation methods (P1-P4), inventory control records (P5-P8) and inventory measurement (P9-P12). Profitability included ROA (P13-P16), gross margin (P17-P20), and ROE (P21-P24). All items used 5-point Likert scales (Never = 1 to Always = 5). The complete correspondence is shown in Table X (instrument source: Questionnaires).

### Development and validation of the instrument

The items were adapted from operations/accounting sources and adjusted to the SMEs; content validity was based on expert judgement (CVI/CVR). A pilot test was conducted to assess clarity and timeliness. Reliability and dimensionality were assessed using Cronbach’s alpha and EFA (KMO, Bartlett); where possible, CFA was estimated and composite reliability (CR) and average variance extracted (AVE) were calculated (criteria CR ≥ 0.70; AVE ≥ 0.50).

### Measurement properties

Internal consistency was acceptable to excellent: α (DIM1) = 0.749; α (DIM2) = 0.858; α (DIM3) = 0.842; α (DIM4) = 0.888; α (DIM5) = 0.798; α (DIM6) = 0.829; sampling adequacy supported factor analysis (IV: KMO = 0.866; DV: KMO = 0.913; all items: KMO = 0.910); Bartlett’s tests rejected sphericity (IV: χ ^2^ = 1111.59, df = 66; DV: χ ^2^ = 1432.63, df = 66; all items: χ ^2^ = 2818.27, df = 276).

### Bias mitigation and diagnosis

Procedural corrective measures included anonymity, balanced wording, separate blocks, and a multiple informant approach; statistically, a Harman factor test was performed as exploratory screening, a marker variable adjustment, and, subject to the sample, MTMM/CFA.

### Statistical analysis plan

Assumption checks (Shapiro-Wilk, Levene) preceded inference. Main models used OLS with industry/size/age controls; robustness checks included alternative specifications, exclusion of influential cases, and marker-adjusted correlations.

## Results and discussion

### Descriptive results

Descriptive statistics for the inventory management and profitability variables are presented in Table 1; overall performance was moderate across all dimensions, with inventory measurement receiving the highest mean score (M = 2.62), indicating an operational emphasis on tracking turnover and days of inventory. The average score of 3 across most variables reinforces a prevailing level of acceptable performance among the micro and small enterprises surveyed; a negative skew was observed across all dimensions, particularly in profitability metrics such as ROA (sk = -0.947) and GM (sk = -1.007), indicating a tendency for profitability to be lower than expected. A negative asymmetry was observed across all dimensions, particularly in profitability metrics such as ROA (sk = -0.947) and GM (sk = -1.007), suggesting that most firms are performing relatively well, while a smaller subset with poor performance is dragging down the mean. These results highlight that companies show greater maturity in metrics and monitoring than in planning and valuation, in line with studies in which MSMEs adopt visible control tools before strengthening their accounting bases ( Ahmad et al., 2022; Sultan, 2021).

**Table 1.** Multidimensional statistical characterization of inventory management and profitability indicators.

Variables		IC	IVM	ICR	IM	P	ROA	GM	ROE
N	Valid	200	200	200	200	200	200	200	
	Lost	0	0	0	0	0	0	0	
Mean		2,37	2,40	2,50	2,62	2,44	2,49	2,55	2,54
Median		2,00	2,50	3,00	3,00	2,50	3,00	3,00	3,00
Mode		2	3	3	3	3	3	3	3
Standard deviation		,619	,672	,665	,647	,599	,665	,608	,625
Variance		,384	,451	,442	,419	,359	,442	,369	,390
Skewness		-,434	-,665	-,965	-1,448	-,565	-,947	-1,007	-1,027
Standard		,172	,172	,172	,172	,172	,172	,172	,172
Kurtosis		-,650	-,633	-,228	,833	-,594	-,257	,003	,001
Standard error of Skewness		,342	,342	,342	,342	,342	,342	,342	,342
Rango		2	2	2	2	2	2	2	2
Minimum		1	1	1	1	1	1	1	1
Maximum		3	3	3	3	3	3	3	3

### Regression analysis

The variables in Table 2 Inventory measurement was the strongest indicator and the only robust predictor of profitability (β = 0.383; p = 0.001), highlighting that companies capable of effectively tracking turnover, days of inventory, and obsolescence costs strengthen their margins and returns. This is consistent with Vahdani and Sazvar (2022), who argue that monitoring real-time inventory dynamics reduces capital immobilisation and improves ROA/ROE.

**Table 2.** Regression model summary, ANOVA, and standardized coefficients.

A. Regression Model and ANOVA	
Estadístico	Valor
R	0.634
R ^2^	0.402
R ^2^ Ajustado	0.390
Error estándar de la estimación	0.468
F	32.81
gl (Regresión/Residual)	4/195
Sig. F	0.001
Durbin-Watson	1.525

B. Model coefficients						
Predictor	B	SE	β	t	Sig.	VIF
(Constant)	0.941	0.201	—	4.68	0.001	—
Inventory Control (IC)	0.198	0.079	0.257	2.50	0.013	1.74
Inventory Valuation Methods (IVM)	0.039	0.049	0.053	0.78	0.436	1.66
Inventory Control Records (ICR)	0.026	0.060	0.037	0.44	0.663	1.82
Inventory Measurement (IM)	0.321	0.064	0.383	5.04	0.001	1.55

Model fit.

R = 0.634; R ^2^ = 0.402; adjusted R ^2^ = 0.390.

F(4,195) = 32.81; p < 0.001.

Durbin-Watson = 1.525 → no autocorrelation issues.

VIF < 2 → no multicollinearity issues.

Structural representation of effects.

**Figure 1.**
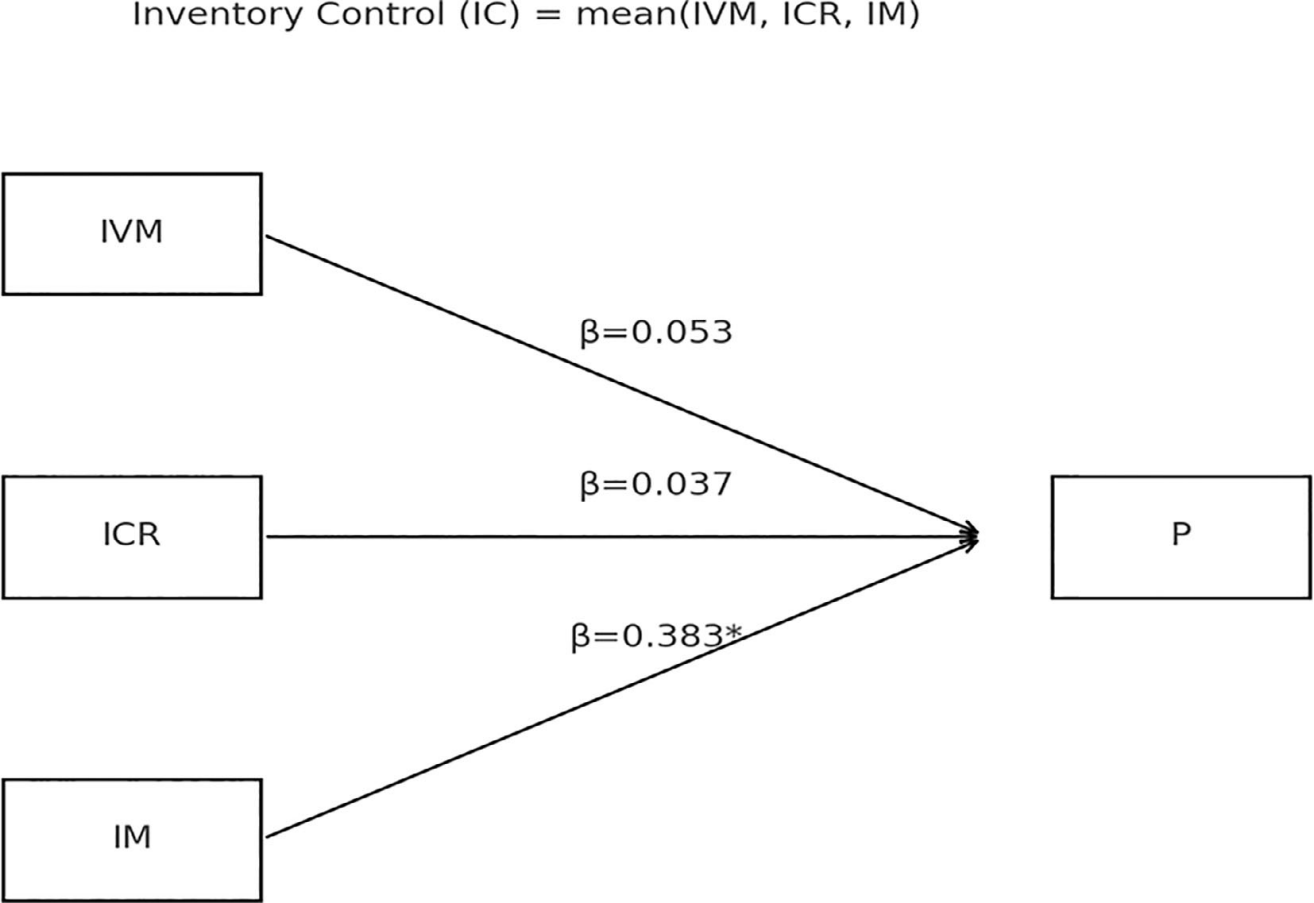
Conceptual model of inventory-management effects on profitability (standardized coefficients).

Inventory control also showed a statistically significant effect (β = 0.257; p = 0.013), suggesting that replenishment policies, safety stock, and order tracking help companies avoid stockouts and reduce emergency purchases, in line with Wilson (2023) and Panigrahi A, et al. (2024).

In contrast, inventory valuation methods and control records showed no direct statistical effects (p > 0.40), supporting contingency-based views: valuation decisions produce indirect effects mediated by the costs of goods sold (IAS 2; Chen and Kong, 2019); records may help internally, but they require integration with planning systems to be reflected in profitability ( Becerra et al., 2022). These findings reinforce the dynamic capabilities approach: capabilities must not only exist, but must be strategically deployed to convert operational data into economic benefits.

The model illustrates that inventory measurement (IM) has the strongest and only statistically significant relationship with profitability (β = 0.383, p < 0.001); MSEs that continuously evaluate turnover, ageing, stockouts, and replenishment rates demonstrate greater operational responsiveness, leading to better cost control and higher financial returns.

In contrast, inventory valuation methods (IVM) (β = 0.053, p > 0.05) and inventory control records (ICR) (β = 0.037, p > 0.05) show no significant direct effect on profitability; these areas, while relevant to accounting accuracy and compliance, appear insufficient on their own to drive financial results without being connected to tactical decision-making.

These data reinforce the idea that measurement capability is a dynamic capability that strengthens strategic decision-making in purchasing and replenishment; in line with the resource-based view (RBV), inventory management becomes a competitive resource when it generates financial benefits and cannot be easily imitated by competitors.

The results are also consistent with contingency theory, which indicates that operational practices must adapt to the environmental uncertainty typical of micro and small enterprises in Bagua, where high demand variability and limited administrative experience increase vulnerability to financial losses.

From a management perspective, the model suggests that micro-enterprises should prioritise inventory analysis dashboards over purely documentary control. In addition, they should strengthen forecasting, reorder points and safety stock policies related to measured KPIs. Similarly, they should integrate IVM and ICR into pricing, purchasing, and logistics workflows to unlock their indirect effect on returns.

## Conclusions

This study provides empirical evidence that inventory measurement and control are the most relevant internal capabilities influencing the profitability of micro and small enterprises (MSEs) in Bagua; organisations that actively monitor stock turnover, days of inventory on hand, and obsolescence risks, and that implement periodic replenishment policies, tend to achieve better financial results, including improved ROA, gross margin, and ROE.

In contrast, inventory valuation methods and record-keeping practices did not show significant direct effects on profitability, suggesting that such practices may contribute indirectly and that their positive financial impact depends on complementary administrative capabilities, such as integration with cost control, demand forecasting, and pricing decisions.

The findings should be interpreted taking into account the contextual limitations of the study: non-probabilistic sampling, self-reported measures, and a restricted geographical scope focused on micro-enterprises in Bagua, Peru; these factors limit the external validity of the results, which should not be generalised to other sectors or regions without caution.

Despite these limitations, the results offer relevant implications for management, such as strengthening digital inventory monitoring, improving staff training in performance metrics (e.g., turnover, GMROI), and incorporating affordable technological solutions (basic ERP/WMS tools) can help MSEs improve operational visibility and reduce pressures on working capital. Policy makers and business support programmes can also leverage these insights to promote capacity-building strategies focused on financial efficiency.

Future research should explore probability-based sampling, triangulation with accounting and enterprise resource planning (ERP) records to reduce common method bias, and SEM-PLS modelling to examine indirect paths and confirm proposed causal mechanisms. such studies would help validate whether the relationships observed in Bagua hold in broader contexts, contributing to the practical and theoretical development of inventory management in emerging economies.

### Ethics and consent

This research was approved by the Ethics Committee of César Vallejo University (Resolution No. 447-2023-VI-UCV of the Vice-Rectorate for Research), in accordance with the principles of the Declaration of Helsinki. All procedures complied with national and international ethical standards applicable to research involving human participants.

Written informed consent was obtained from all participants before administering the questionnaire. All respondents were over 18 years of age and were fully informed about the objectives of the study, the voluntary nature of their participation, the confidentiality of their data, and the exclusive use of the information for academic and scientific purposes.

## Data Availability

All data supporting the results of this study including the values underlying the reported means, standard deviations, and other measures; the values used to generate the figures; and the data points extracted from images for analysis are available in the Data availability the Victor Puican repository. **Process identifier** https://doi.org/10.23728/B2SHARE.C7BA1BDAF7B8423A8D0E467A446EB0F9 ( Rodriguez et al., 2025). **Additional materials and data** The additional data used in this study, including the complete questionnaire, instrument application guide, and supplementary tables of statistical results, are publicly available in the same data repository. **Process identifier** https://doi.org/10.23728/B2SHARE.C7BA1BDAF7B8423A8D0E467A446EB0F9 ( Rodriguez et al., 2025). These materials allow for open replication and review of the study. The full dataset is accessible without restrictions or embargoes under a CC-BY 4.0 license.
