# Innovations in Inventory Management to Improve the Profitability of Local SMEs

**DOI:** 10.12688/f1000research.168900.3

**Published:** 2025-11-29

**Authors:** Víctor Hugo Puican Rodríguez, RITA DE JESUS TORO LÓPEZ, Waldemar Ramón García Vera

**Affiliations:** 1 Universidad Cesar Vallejo, Trujillo, La Libertad, Peru; 2 Universidad Cesar Vallejo, Trujillo, La Libertad, Peru; 3 Universidad Cesar Vallejo, Trujillo, La Libertad, Peru

**Keywords:** Inventory, profitability, control, measurement, financial efficiency.

## Abstract

**Background:**

Efficient inventory management is a critical internal capability for ensuring the financial sustainability of micro and small enterprises, especially in emerging economies affected by post-pandemic disruptions. In Bagua, Peru, MSMEs often lack digitised control systems, leading to frequent stock imbalances, rushed purchases at high prices, and lower profitability; a deeper understanding of inventory practices that drive financial performance can contribute to more informed and strategic decision-making.

**Method:**

This quantitative, descriptive, and explanatory study examined the effects of four components of inventory management—control, valuation methods, control records, and measurement—on profitability; a convenience sample of 83 MSMEs yielded 200 valid responses from key personnel involved in inventory decisions; A 21-item Likert scale validated by experts was applied, and the data were analysed using descriptive statistics and multiple linear regression in SPSS v27. Profitability indicators included return on assets (ROA), gross margin (GM), and return on equity (ROE).

**Results:**

All dimensions of the inventory showed moderate levels of implementation (means: 2.37–2.62 on a five-point scale). The regression model demonstrated satisfactory predictive power (R ^2^ = 0.402; F = 32.81; p < 0.001). Inventory measurement was the strongest and only highly significant predictor of profitability (β = 0.383; p < 0.001), followed by inventory control, with a smaller but significant contribution (β = 0.257; p = 0.013). Inventory valuation methods and control records did not show statistically significant direct effects (p > 0.40).

**Conclusion:**

The findings highlight that decision-oriented measurement practices, supported by systematic control, are essential factors for the financial performance of micro and small enterprises; investment in digital monitoring tools, replenishment based on key performance indicators, and staff training could improve operational and financial efficiency; due to its cross-sectional design and localised context, future studies should incorporate longitudinal data and broader geographical comparisons to strengthen generalisation and explore possible mediation pathways between accounting-oriented practices and profitability.

## Introduction

Efficient inventory management is one of the most influential operational capabilities in the financial performance of micro and small enterprises (MSEs). Beyond controlling losses, avoiding stockouts, or reducing obsolescence, inventory decisions directly affect working capital discipline and profitability through indicators such as days of inventory (DIO), cash conversion cycle (CCC), and margin metrics related to inventory turnover and profitability ( Ranjan et al., 2024). Several recent reviews agree that when these practices are complemented by accessible technologies and appropriate management criteria, SMEs can improve their responsiveness and optimise the use of resources in environments characterised by financial and operational constraints.

On a technical level, replenishment policies under continuous review (R, Q), together with demand classification and monitoring of stockout risks, are key to maintaining optimal inventory levels and controlling maintenance costs. In situations of intermittent or irregular demand common in local assortments evidence shows that Croston-type methods and their corrected variants tend to offer superior performance compared to simple smoothing techniques, especially for low-turnover products ( Zhou, 2023; Wilson et al., 2022; Wilson, 2023). At the same time, the progressive adoption of digital inventory tools is transforming the competitive landscape: new companies with greater technological capabilities are putting pressure on established businesses, and failure to adapt in a timely manner can weaken key operational advantages ( Thomas & Douglas, 2024; Asad et al., 2025; Ni et al., 2024).

From an accounting perspective, the way inventories are valued and recorded determines the cost of sales, margin structure, and reliability of financial statements; the IFRS/IAS 2 framework sets strict guidelines on permitted methods FIFO and weighted average and demands consistency, accuracy, and proper recognition of impairment; however, in small businesses, the application of these standards depends largely on the availability of recording systems, the level of formalisation, and the experience of the personnel in charge, factors that can limit the translation of accounting practices into concrete improvements in profitability.

### Research gap

Despite the importance of these practices, the available empirical evidence focuses on medium-sized or large companies, usually located in urban or industrial contexts. Little is known about how these relationships manifest themselves in MSMEs in geographically dispersed areas, where infrastructure limitations, informality, and variability in delivery times can modify the expected effects of inventory management. In the Peruvian Amazon, 96% of business units are microenterprises, predominantly engaged in commercial activities with high turnover and low margins ( Ministry of Production, 2024). These characteristics mean that seemingly simple decisions such as defining reorder points, keeping accurate records, or adequately measuring turnover have a particularly sensitive impact on their financial performance.

### Relevance of the Bagua case

Bagua is a representative setting for studying this issue; the heterogeneity of its road infrastructure, connectivity limitations, and variability in supply times create a logistical context that differs from that of the country's coastal or metropolitan areas. Under these conditions, optimal replenishment parameters, safety stock levels, and inventory measurement policies may differ significantly, providing an analytical opportunity to understand how these practices are adjusted in environments with greater operational constraints ( Suárez et al., 2024). Likewise, studying Bagua provides relevant evidence for other intermediate cities in the Amazon and Latin America with similar characteristics.

### Contribution of the study

This study quantifies the relationship between specific inventory management practices—control, valuation, and measurement and the profitability of micro and small enterprises located in Bagua, incorporating criteria aligned with IFRS/IAS 2. The analysis identifies which operational capabilities have a real financial impact in environments where the business structure is fragile and resources are limited. By addressing a poorly researched area and connecting operational and accounting perspectives, the study contributes to understanding how simple but consistent practices can generate tangible economic benefits and improve the financial stability of MSMEs in highly vulnerable contexts ( KPMG, 2021).

## Literature review

### Theoretical framework: RBV approach and dynamic capabilities

Inventory management is a fundamental operational capability within the resource-based view (RBV) framework: organisations reap financial benefits when internal resources, such as inventory control processes, ICT systems and forecasting skills, create cost advantages or improve responsiveness ( Barney, 1991; El Nemar et al., 2022). From a dynamic capabilities perspective, the ability to detect fluctuations in demand, take advantage of replenishment opportunities and reconfigure stock policies is particularly important for micro and small enterprises exposed to volatile environments, such as the Amazon region of Peru ( Teece, 2007; Chen et al., 2024). Recent studies indicate that these capabilities are being integrated with Industry 4.0 tools such as artificial intelligence, the Internet of Things, and advanced analytics, strengthening both competitive advantage and sustainability outcomes and providing updated theoretical support for RBV/DC arguments.

### Post-COVID context and restrictions on MSMEs

In the post-COVID-19 pandemic scenario, efficient inventory management has become a decisive factor for the operational and financial sustainability of MSMEs in Peru ( Carpitella & Izquierdo, 2025). The adoption of appropriate stock control and measurement strategies can generate tangible benefits by minimising losses due to expiry, deterioration or theft of products ( Lefebvre, 2025). However, a significant proportion of these organisations continue to operate without digitised systems that allow for real-time monitoring, which limits the quality of decisions and reduces profitability ( Odumusor, 2024). MSMEs require flexibility in inventory management due to demand variability and price sensitivity ( Ranjan et al., 2024).

However, many face difficulties associated with the inexperience of their managers and low shareholder involvement, affecting key indicators such as return on assets (ROA) and return on equity (ROE) ( Issah et al., 2024). Likewise, the absence of safety stock policies leads to emergency purchases at high prices, increasing the cost of products and negatively affecting sales levels ( Chelestino et al., 2024).

Empirical evidence from Latin America and other regions of the Global South concurs with these limitations, documenting manual and empirical inventory practices in numerous micro and small businesses, and highlighting the positive impacts generated even by basic digitisation processes, such as the implementation of ERP, WMS, barcodes, RFID or scorecards.

### Definitions, mechanisms and operational fundamentals

Inventory control refers to coordinated decisions about the timing and quantity of replenishment, commonly represented by continuous review policies (R, Q), which determine stock-out costs, carrying costs, and service levels ( Gavhane, 2025; Ramadan et al., 2024). Basic profitability channels operate through days of inventory outstanding (DIO), cash conversion cycle (CCC), and inventory profitability ratios such as GMROI ( Maheshwari et al., 2025). Safety stock policies mitigate delivery time uncertainty but must balance capital tie-up and service performance ( Kotecha & del Rio, 2025).

Recent contributions have modelled these policies by combining classical approaches such as EOQ or (s, S) with metaheuristics and dynamic systems, demonstrating substantial improvements in costs and service levels that impact working capital and margins. Likewise, accounting valuation aligned with IFRS, particularly IAS 2, continues to provide transparency in margin measurement by applying methods such as FIFO or weighted average.

### Empirical evidence and methodological approaches

Current studies have revealed a consistent positive relationship between CI practices and the profitability of micro and small enterprises, especially those where management skills and digital visibility remain scarce ( Li and Mizuno, 2022; Panigrahi et al., 2024b). In terms of replenishment decisions, EOQ-type models and their extensions remain viable, low-cost solutions for companies with limited analytical capabilities, as they reduce ordering and storage costs ( Condeixa et al., 2020; Çalışkan, 2021). However, demand in small retailers is often intermittent or irregular, favouring Croston-type methods over naive smoothing to avoid systematic biases and service losses ( Syntetos et al., 2005; Erjiang et al., 2022).

Accurate record keeping and IFRS-aligned valuation methods (IAS 2), i.e. FIFO or weighted average, improve cost allocation, margin visibility and the quality of financial reporting ( Ala et al., 2023). Similarly, KPIs such as compliance rate, inventory turnover, and shrinkage rate have proven to be highly predictive of operating margins ( Sánchez-Córdova et al., 2024).

Therefore, integrated inventory control systems, whether manual or digital, impact profitability not only through cost containment but also through strategic differentiation, especially in short-life-cycle categories susceptible to deterioration ( Maolani et al., 2025).

Despite these data, geographical coverage remains skewed towards the urban and well-digitised ecosystems of Asia and Europe, with limited evidence in secondary cities or Amazonian economies ( Granillo-Macías et al., 2024). The structural conditions of these environments informality, long delivery times, concentration of suppliers may moderate the link between inventory policies and profitability ( Singh et al., 2022). This contingency suggests that practices that are effective in metropolitan contexts may not produce similar effects in regions such as Bagua, Peru. The dynamism that exists in companies is an extraordinary capacity linked to the innovation necessary for the implementation of eco-friendly product innovation ( Asad et al., 2024; Lütjen et al. 2019).

Due to the ongoing diversity of the supply chain and the increased risks faced by small and medium-sized enterprises, they tend to be overly concerned about risks, as they have little capacity to cope with major disruptions. Added to this is the scarcity of resources they possess. the moderating role of business networks and the current pace of technological change have had a serious impact on management, leading to reduced profitability in various economic periods ( Awain et al., 2025).

The data obtained provides a better understanding of the relationship between supplier relationship management (SCRM) and innovative performance in MSMEs. Specifically, it provides empirical evidence on the impact of SCRM components, such as maturity and capability, on innovative performance. It also shows the impact of technological turbulence on both SCRM and innovative performance ( Foli et al., 2024; Sulaiman et al., 2024). Furthermore, a significant relationship was found between green entrepreneurial orientation, green market orientation, and green learning orientation with the sustainable performance of small and medium-sized enterprises (SMEs) in the textile sector ( Uzair & Abu, 2025). At the same time, they pointed out that a data-driven organisational culture has a considerable moderating impact on product innovation and process improvement, which ultimately enhances business value through better overall company performance ( Chaudhuri et al., 2024).

Adaptability acts as a mediator in the association between commercial ties and business performance. At the same time, they indicate that the indirect effect of commercial ties on business performance, through adaptability, was moderated by technological turbulence ( Uzkurt et al., 2024; Satar et al., 2024). Several studies have shown that SMEs can improve their performance when they innovate in green products and processes. A significant moderating variable in SME performance is technological turbulence, thus contributing to a better understanding of green innovation and having implications for the design of policies that promote green development ( Ta'amnha et al., 2024; Monze & Chibolo, 2025; Kisinga et al., 2024).

Recent studies document precisely these barriers, highlighting the predominance of manual controls, limited data capture, and long replenishment times. They also show that the gradual adoption of ERP, WMS, and lean warehouse practices generates verifiable improvements in compliance rates, inventory turnover, and operating margins, providing external validity for Amazonian MSMEs and underscoring the need for context-sensitive designs.

### Summary and gap

Previous studies corroborate that inventory capabilities create financial value, but few studies integrate RBV with dynamic capabilities to explain profitability outcomes in SMEs under the specific constraints of the Amazon; Consequently, this study examines how inventory control and measurement practices affect ROA, GM, and ROE in Bagua-based SMEs, addressing a geographical and theoretical gap with practical implications for similar contexts in developing economies.

## Methods

### Research design

A quantitative descriptive-explanatory design was used, which is appropriate for examining the relationship between inventory management capabilities and business profitability. This approach made it possible to evaluate the direct effects of three operational dimensions (valuation methods, control records and inventory measurement) on financial indicators (ROA, gross margin and ROE) using multiple linear regression models. The choice of OLS analysis was justified by the explanatory objective of the study and the continuous nature of the variables, as well as by the finding that inventory measurement is the most robust predictor of profitability (β = 0.383; p = 0.001).

### Population and sample

The population consisted of formally registered and active micro and small enterprises (MSEs) in the province of Bagua (Peru) with five or more employees. Eighty-three companies in the commerce/services and construction sectors were contacted, inviting five informants per unit (manager, warehouse manager, accountant, and two accounting assistants) to minimise single-informant bias. The inclusion criteria considered active tax registration, operational continuity during the last two years, and willingness to participate. The final valid sample consisted of 200 informants from 40 enterprises.

### Sampling strategy

Non-probability convenience sampling was used due to access restrictions and management authorisation. This approach may limit generalisation; however, it is appropriate in contexts where MSMEs are highly sensitive about disclosing operational information. The descriptive results obtained including the marked negative asymmetry in profitability indicators suggest that future research should use stratified probability sampling and longitudinal designs.

### Variables and operationalisation

Inventory management was structured into three dimensions:

Valuation methods (P1–P4);

Control records (P5–P8);

Inventory measurement (P9–P12).

### Profitability included

ROA (P13–P16),

Gross margin (P17–P20),

ROE (P21–P24).

All items used a 5-point Likert scale (1 = Never; 5 = Always). Descriptive analysis showed moderate performance (means between 2.37 and 2.62), justifying the application of inferential methods to identify significant predictors.

### Validity and reliability of the instrument

The items were adapted from specialised literature on operations and accounting and adjusted to the context of MSMEs. Content validity was ensured through expert judgement using CVR and CVI. The pilot test confirmed the clarity and consistency of the instrument.

Exploratory and confirmatory factor analyses were performed. The sample adequacy indices were satisfactory: KMO inventories = 0.866; KMO profitability = 0.913; KMO overall = 0.910; Bartlett p < 0.001. Internal reliability was acceptable to excellent (α between 0.749 and 0.888). In the confirmatory analysis, the constructs met the criteria for composite reliability (CR ≥ 0.70) and convergent validity (AVE ≥ 0.50).

### Bias control

To mitigate the bias of the common method, procedural measures were implemented (anonymity, separate blocks, balanced wording, and participation of multiple informants). Additionally, statistical tests were applied: Harman's single-factor test, marker variable, and, when possible, MTMM model.

### Pre-analysis tests

Before estimating the models, the following assumptions were verified:

Normality using Shapiro–Wilk (with moderate deviations tolerable given n = 200); Homoscedasticity using Levene's test; Collinearity ruled out (VIF < 2); Independence of errors confirmed (Durbin–Watson = 1.525). These results support the technical relevance of the OLS model applied.

### Analysis model

A multiple linear regression was estimated with controls for sector, size, and age. The results showed that inventory measurement was the strongest and most consistent predictor of profitability (β = 0.383; p = 0.001), followed by inventory control (β = 0.257; p = 0.013). Valuation methods and control records did not show significant direct effects (p > 0.40), which is consistent with contingency approaches and with the literature indicating that these practices generate indirect effects when integrated into tactical purchasing, replenishment, and pricing decisions.

## Results and discussion

### Descriptive results

Descriptive statistics for the inventory management and profitability variables are presented in Table 1; overall performance was moderate across all dimensions, with inventory measurement receiving the highest mean score (M = 2.62), indicating an operational emphasis on tracking turnover and days of inventory. The average score of 3 across most variables reinforces a prevailing level of acceptable performance among the micro and small enterprises surveyed; a negative skew was observed across all dimensions, particularly in profitability metrics such as ROA (sk = -0.947) and GM (sk = -1.007), indicating a tendency for profitability to be lower than expected. A negative asymmetry was observed across all dimensions, particularly in profitability metrics such as ROA (sk = -0.947) and GM (sk = -1.007), suggesting that most firms are performing relatively well, while a smaller subset with poor performance is dragging down the mean. These results highlight that companies show greater maturity in metrics and monitoring than in planning and valuation, in line with studies in which MSMEs adopt visible control tools before strengthening their accounting bases ( Ahmad et al., 2022; Sultan, 2021).

**Table 1.** Multidimensional statistical characterization of inventory management and profitability indicators.

Variables		IC	IVM	ICR	IM	P	ROA	GM	ROE
N	Valid	200	200	200	200	200	200	200	
	Lost	0	0	0	0	0	0	0	
Mean		2,37	2,40	2,50	2,62	2,44	2,49	2,55	2,54
Median		2,00	2,50	3,00	3,00	2,50	3,00	3,00	3,00
Mode		2	3	3	3	3	3	3	3
Standard deviation		,619	,672	,665	,647	,599	,665	,608	,625
Variance		,384	,451	,442	,419	,359	,442	,369	,390
Skewness		-,434	-,665	-,965	-1,448	-,565	-,947	-1,007	-1,027
Standard		,172	,172	,172	,172	,172	,172	,172	,172
Kurtosis		-,650	-,633	-,228	,833	-,594	-,257	,003	,001
Standard error of Skewness		,342	,342	,342	,342	,342	,342	,342	,342
Rango		2	2	2	2	2	2	2	2
Minimum		1	1	1	1	1	1	1	1
Maximum		3	3	3	3	3	3	3	3

### Regression analysis

The variables in Table 2 Inventory measurement was the strongest indicator and the only robust predictor of profitability (β = 0.383; p = 0.001), highlighting that companies capable of effectively tracking turnover, days of inventory, and obsolescence costs strengthen their margins and returns. This is consistent with Vahdani and Sazvar (2022), who argue that monitoring real-time inventory dynamics reduces capital immobilisation and improves ROA/ROE.

**Table 2.** Regression model summary, ANOVA, and standardized coefficients.

A. Regression Model and ANOVA	
Estadístico	Valor
R	0.634
R ^2^	0.402
R ^2^ Ajustado	0.390
Error estándar de la estimación	0.468
F	32.81
gl (Regresión/Residual)	4/195
Sig. F	0.001
Durbin-Watson	1.525

B. Model coefficients						
Predictor	B	SE	β	t	Sig.	VIF
(Constant)	0.941	0.201	—	4.68	0.001	—
Inventory Control (IC)	0.198	0.079	0.257	2.50	0.013	1.74
Inventory Valuation Methods (IVM)	0.039	0.049	0.053	0.78	0.436	1.66
Inventory Control Records (ICR)	0.026	0.060	0.037	0.44	0.663	1.82
Inventory Measurement (IM)	0.321	0.064	0.383	5.04	0.001	1.55

Model fit.

R = 0.634; R ^2^ = 0.402; adjusted R ^2^ = 0.390.

F(4,195) = 32.81; p < 0.001.

Durbin-Watson = 1.525 → no autocorrelation issues.

VIF < 2 → no multicollinearity issues.

Structural representation of effects.

**Figure 1.**
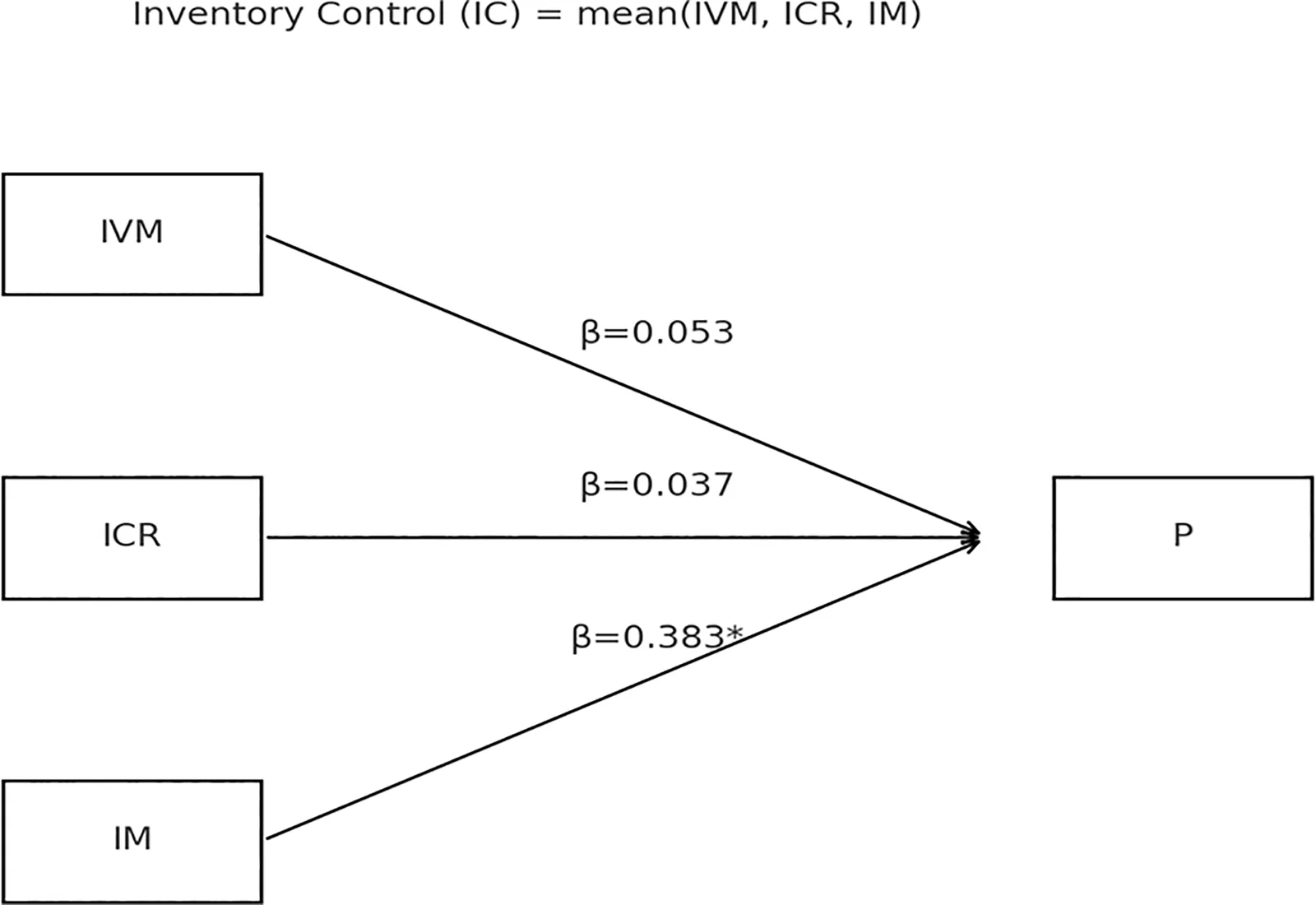
Conceptual model of inventory-management effects on profitability (standardized coefficients).

Inventory control also showed a statistically significant effect (β = 0.257; p = 0.013), suggesting that replenishment policies, safety stock, and order tracking help companies avoid stockouts and reduce emergency purchases, in line with Wilson (2023) and Panigrahi et al. (2024a).

In contrast, inventory valuation methods and control records showed no direct statistical effects (p > 0.40), supporting contingency-based views: valuation decisions produce indirect effects mediated by the costs of goods sold (IAS 2; Chen and Kong, 2019); records may help internally, but they require integration with planning systems to be reflected in profitability (Becerra et al., 2022). These findings reinforce the dynamic capabilities approach: capabilities must not only exist, but must be strategically deployed to convert operational data into economic benefits.

The model illustrates that inventory measurement (IM) has the strongest and only statistically significant relationship with profitability (β = 0.383, p < 0.001); MSEs that continuously evaluate turnover, ageing, stockouts, and replenishment rates demonstrate greater operational responsiveness, leading to better cost control and higher financial returns.

In contrast, inventory valuation methods (IVM) (β = 0.053, p > 0.05) and inventory control records (ICR) (β = 0.037, p > 0.05) show no significant direct effect on profitability; these areas, while relevant to accounting accuracy and compliance, appear insufficient on their own to drive financial results without being connected to tactical decision-making.

These data reinforce the idea that measurement capability is a dynamic capability that strengthens strategic decision-making in purchasing and replenishment; in line with the resource-based view (RBV), inventory management becomes a competitive resource when it generates financial benefits and cannot be easily imitated by competitors.

The results are also consistent with contingency theory, which indicates that operational practices must adapt to the environmental uncertainty typical of micro and small enterprises in Bagua, where high demand variability and limited administrative experience increase vulnerability to financial losses.

From a management perspective, the model suggests that micro-enterprises should prioritise inventory analysis dashboards over purely documentary control. In addition, they should strengthen forecasting, reorder points and safety stock policies related to measured KPIs. Similarly, they should integrate IVM and ICR into pricing, purchasing, and logistics workflows to unlock their indirect effect on returns.

## Conclusions

This study provides empirical evidence that inventory measurement and control are the most relevant internal capabilities influencing the profitability of micro and small enterprises (MSEs) in Bagua; organisations that actively monitor stock turnover, days of inventory on hand, and obsolescence risks, and that implement periodic replenishment policies, tend to achieve better financial results, including improved ROA, gross margin, and ROE.

In contrast, inventory valuation methods and record-keeping practices did not show significant direct effects on profitability, suggesting that such practices may contribute indirectly and that their positive financial impact depends on complementary administrative capabilities, such as integration with cost control, demand forecasting, and pricing decisions.

The findings should be interpreted taking into account the contextual limitations of the study: non-probabilistic sampling, self-reported measures, and a restricted geographical scope focused on micro-enterprises in Bagua, Peru; these factors limit the external validity of the results, which should not be generalised to other sectors or regions without caution.

Despite these limitations, the results offer relevant implications for management, such as strengthening digital inventory monitoring, improving staff training in performance metrics (e.g., turnover, GMROI), and incorporating affordable technological solutions (basic ERP/WMS tools) can help MSEs improve operational visibility and reduce pressures on working capital. Policy makers and business support programmes can also leverage these insights to promote capacity-building strategies focused on financial efficiency.

Future research should explore probability-based sampling, triangulation with accounting and enterprise resource planning (ERP) records to reduce common method bias, and SEM-PLS modelling to examine indirect paths and confirm proposed causal mechanisms. such studies would help validate whether the relationships observed in Bagua hold in broader contexts, contributing to the practical and theoretical development of inventory management in emerging economies.

### Ethics and consent

This research was approved by the Ethics Committee of César Vallejo University (Resolution No. 447-2023-VI-UCV of the Vice-Rectorate for Research), in accordance with the principles of the Declaration of Helsinki. All procedures complied with national and international ethical standards applicable to research involving human participants.

Written informed consent was obtained from all participants before administering the questionnaire. All respondents were over 18 years of age and were fully informed about the objectives of the study, the voluntary nature of their participation, the confidentiality of their data, and the exclusive use of the information for academic and scientific purposes.

## Data Availability

All data supporting the results of this study including the values underlying the reported means, standard deviations, and other measures; the values used to generate the figures; and the data points extracted from images for analysis are available in the Data availability the Victor Puican repository. **Process identifier** https://doi.org/10.23728/B2SHARE.C7BA1BDAF7B8423A8D0E467A446EB0F9 ( Rodriguez et al., 2025). **Additional materials and data** The additional data used in this study, including the complete questionnaire, instrument application guide, and supplementary tables of statistical results, are publicly available in the same data repository. **Process identifier** https://doi.org/10.23728/B2SHARE.C7BA1BDAF7B8423A8D0E467A446EB0F9 ( Rodriguez et al., 2025). These materials allow for open replication and review of the study. The full dataset is accessible without restrictions or embargoes under a CC-BY 4.0 license.
