# Supplementary material for: Innovations in Inventory Management to Improve the Profitability of Local SMEs
Source: F1000Res. 2025 Nov 29;14:853. Originally published 2025 Sep 2. [Version 3] doi: 10.12688/f1000research.168900.3 (PMC12754351; doi:10.12688/f1000research.168900.3)
Supplement: Peer review history

# Reviewer report: Reviewer response for version 3

**DOI:** 10.5256/f1000research.191965.r437716

**Published:** 2026-10-20

**Authors:** Embun Suryani

**Affiliations:** 1 University of Mataram, Mataram, Indonesia

The manuscript investigates the relationship between inventory management practices and profitability among micro and small enterprises in Bagua, Peru. Using questionnaire responses and multiple regression, the authors report that inventory measurement and inventory control are positively associated with profitability, whereas valuation methods and control records are not statistically significant. The topic has practical relevance, but substantial inconsistencies in measurement, sampling, and model specification currently prevent confidence in the findings.

**1. Clarity, accuracy, and literature — Partly**

 The article has a recognisable structure and cites recent literature. However, important inconsistencies undermine its accuracy. The abstract refers to 83 enterprises and 21 questionnaire items, whereas the methods describe 200 respondents from 40 enterprises and 24 items. The manuscript also alternates between three and four inventory-management dimensions.

 Some citations appear unrelated to the claims they support. For example, Wilson et al. (2022), listed as a study of molecular solvation, is cited in the discussion of inventory forecasting.

**Required:** Reconcile the sample, instrument, and construct descriptions throughout the manuscript. Audit all citations for relevance and accuracy, and strengthen the synthesis connecting the literature to the variables actually measured.

**2. Study design and technical soundness — No**

 A cross-sectional survey can examine associations, but the unit of analysis is unclear. The methods indicate five informants per enterprise, yet the regression treats 200 responses as observations without explaining how dependence within enterprises was handled. This may affect standard errors and significance tests.

 Profitability is measured using Likert items labelled ROA, gross margin, and ROE. These responses cannot be interpreted as actual accounting ratios without supporting financial records.

**Required:** Identify the final number of enterprises and respondents, explain respondent selection, and clarify whether the analysis concerns individuals or enterprises. Address clustering through an appropriately justified approach, such as multilevel modelling, cluster-adjusted inference, or enterprise-level aggregation supported by agreement and reliability evidence. Reframe the outcome as perceived profitability unless actual financial ratios were collected.

**3. Replicability — No**

 Essential information remains missing or contradictory. Although a five-point response scale is described, Table 1 reports a maximum of three for every variable. The scoring or recoding process is not explained. Instrument-validation procedures are mentioned, but numerical results are insufficiently reported.

**Required:** Provide the complete questionnaire, item sources, scoring rules, recoding procedures, data-collection dates, exclusion criteria, and missing-data handling. Report the actual factor-analysis and reliability results, including model specification and fit where applicable. Identify which common-method-bias tests were performed and provide their results.

**4. Statistical analysis and interpretation — No**

 The most serious concern is the relationship between the predictors. Figure 1 defines inventory control as the mean of valuation methods, control records, and inventory measurement, while Table 2 includes all four as simultaneous predictors. If that definition was used in the analysis, the predictors are linearly dependent, which is incompatible with the reported low VIF values.

 The methods also claim adjustment for sector, size, and enterprise age, but these controls do not appear in Table 2. The reported regression degrees of freedom indicate four predictors only. Furthermore, Durbin–Watson does not establish independence among respondents within the same enterprise.

**Required:** Clarify whether inventory control is a separate construct or a composite of the other predictors. Correct the model or figure accordingly and rerun the analysis where necessary. Report the complete model, including any controls, coefficients, confidence intervals, diagnostics, and the approach used to address clustering. Verify consistency between the tables, figure, and narrative, including the contradictory statement that inventory measurement is the only significant predictor despite inventory control having p = .013.

**5. Source data and reproducibility — Partly**

 The manuscript provides a repository DOI and states that data and supplementary materials are available. However, the repository contents were not independently verified in this assessment, and the manuscript alone does not establish full reproducibility.

**Required:** Ensure that the repository includes anonymised item-level responses, enterprise identifiers sufficient to reconstruct clustering, a codebook, scoring instructions, the questionnaire, and analysis syntax. Explain how the deposited files reproduce the reported tables and resolve the discrepancies identified above.

**6. Support for the conclusions — Partly**

 The conclusions reflect the reported coefficient directions, but their reliability depends on resolving the analytical concerns. Causal claims about improving profitability exceed the cross-sectional evidence. Nonsignificant coefficients for valuation and record-keeping do not demonstrate indirect effects, because mediation was not tested.

**Required:** Limit conclusions to associations with perceived profitability in the sampled enterprises. Present indirect mechanisms and benefits of digital tools as possible explanations or future research questions, rather than established findings.

**Overall assessment**

 The manuscript requires substantial methodological clarification and potentially reanalysis. The essential requirements for scientific soundness are to resolve the unit of analysis and clustering, reconcile the instrument and sample descriptions, establish a coherent predictor specification, document outcome measurement, and provide reproducible statistical results. These are substantive issues that cannot be addressed through editorial revision alone. In its current form, I cannot confirm that the analysis is technically sound.

Is the work clearly and accurately presented and does it cite the current literature?

Partly

If applicable, is the statistical analysis and its interpretation appropriate?

No

Are all the source data underlying the results available to ensure full reproducibility?

Partly

Is the study design appropriate and is the work technically sound?

No

Are the conclusions drawn adequately supported by the results?

Partly

Are sufficient details of methods and analysis provided to allow replication by others?

No

Reviewer Expertise:

Financial management

I confirm that I have read this submission and believe that I have an appropriate level of expertise to state that I do not consider it to be of an acceptable scientific standard, for reasons outlined above.

# Reviewer report: Reviewer response for version 3

**DOI:** 10.5256/f1000research.191965.r437232

**Published:** 2025-12-30

**Authors:** Vina Sari Yosephine

**Affiliations:** 1 Universitas Pembangunan Nasional Veteran Jakarta, Jakarta, Special Capital Region of Jakarta, Indonesia

This paper has strong potential now, but requires one more serious methodological-theoretical upgrade, especially in this area.

- A proper causal-safe interpretation,
- A clear dynamic-capability construct mapping,
- And at least one mediation/moderation mechanism.

While the study now explicitly refers to the Resource-Based View and Dynamic Capabilities perspectives, these frameworks are weakly integrated into the analytical structure of the study. The constructs of inventory measurement, control, valuation, and record-keeping are not clearly mapped to specific capability mechanisms such as sensing, seizing, and reconfiguring. As a result, the theoretical contribution  is mainly descriptive rather than explanatory. Strengthening this linkage would  enhance the paper’s contribution to the literature on operational capabilities and firm performance.

 A more critical concern relates to construct validity. Profitability is operationalized through Likert-scale perceptual measures labeled as ROA, gross margin, and ROE. These indicators are, by definition, objective financial ratios, and their measurement through subjective perceptions introduces a conceptual ambiguity that weakens the interpretability of the findings. Unless actual accounting data are used, the authors should clearly reframe these variables as perceived or self-reported profitability. Without this clarification, the empirical model risks conflating managerial perceptions with true financial performance.

 Regarding scientific novelty, the core empirical finding: that inventory measurement and control are more influential than valuation and record-keeping for profitability, is consistent with a substantial body of prior work on working capital management, digital visibility, and inventory analytics.

Is the work clearly and accurately presented and does it cite the current literature?

Partly

If applicable, is the statistical analysis and its interpretation appropriate?

Partly

Are all the source data underlying the results available to ensure full reproducibility?

Partly

Is the study design appropriate and is the work technically sound?

Partly

Are the conclusions drawn adequately supported by the results?

Yes

Are sufficient details of methods and analysis provided to allow replication by others?

Partly

Reviewer Expertise:

Supply Chain Management, Logistics, SME Digitalization

I confirm that I have read this submission and believe that I have an appropriate level of expertise to confirm that it is of an acceptable scientific standard, however I have significant reservations, as outlined above.

# Reviewer report: Reviewer response for version 3

**DOI:** 10.5256/f1000research.191965.r437231

**Published:** 2025-12-11

**Authors:** Muzaffar Asad

**Affiliations:** 1 Entrepreneurship, Tecnológico de Monterrey,, Monterrey, Nuevo Leon, Mexico

I believe that Paper has been improved sufficiently.

Is the work clearly and accurately presented and does it cite the current literature?

Partly

If applicable, is the statistical analysis and its interpretation appropriate?

Partly

Are all the source data underlying the results available to ensure full reproducibility?

No

Is the study design appropriate and is the work technically sound?

Yes

Are the conclusions drawn adequately supported by the results?

Partly

Are sufficient details of methods and analysis provided to allow replication by others?

No

Reviewer Expertise:

Entrepreneurship and SMEs performance

I confirm that I have read this submission and believe that I have an appropriate level of expertise to confirm that it is of an acceptable scientific standard.

# Reviewer report: Reviewer response for version 2

**DOI:** 10.5256/f1000research.190753.r431317

**Published:** 2025-11-20

**Authors:** Muzaffar Asad

**Affiliations:** 1 Entrepreneurship, Tecnológico de Monterrey,, Monterrey, Nuevo Leon, Mexico

I have analyzed the paper and I believe that the topic has good potential. However, in my opinion, the entire work is shallow. The introduction is too brief, the literature has ignored almost majority of the relevant recent studies. The methodology is precise and the analysis is also very precise. the topic is good, but every step needs explanation and support of the most relevant and current literature. I am recommending a few studies, but you must enhance your writing to more literature and add at least the review of 30 40 more articles and strengthen introduction with theoretical gaps that your study is trying to fill. Likewise, for the literature, expand methodology, follow the tests needed to support the kind of analysis you are making.

 I am just recommending a few papers related to innovation and profitability, make sure you need to add more studies into it.

 Asad, M., Sulaiman, M. A., Awain, A. M., Alsoud, M., Allam, Z., & Asif, M. U. (2024). Green entrepreneurial leadership, and performance of entrepreneurial firms: Does green product innovation mediates? *Cogent Business & Management, 11*(1), 2355685. doi:https://doi.org/10.1080/23311975.2024.2355685

 Awain, A. M., Asad, M., Sulaiman, M. A., Asif, M. U., & Shanfari, K. S. (2025). Impact of supply chain risk management on product innovation performance of Omani SMEs: Synergetic moderation of technological turbulence and entrepreneurial networking. *Sustainability, 17*(7), 2903. doi:https://doi.org/10.3390/su17072903

 Sulaiman, M. A., Asad, M., Awain, A. M., Asif, M. U., & Shanfari, K. S. (2024). Entrepreneurial marketing and performance: Contingent role of market turbulence. *Discover Sustainability, 5*, 1-20. doi:https://doi.org/10.1007/s43621-024-00710-8

Is the work clearly and accurately presented and does it cite the current literature?

Partly

If applicable, is the statistical analysis and its interpretation appropriate?

Partly

Are all the source data underlying the results available to ensure full reproducibility?

No

Is the study design appropriate and is the work technically sound?

Yes

Are the conclusions drawn adequately supported by the results?

Partly

Are sufficient details of methods and analysis provided to allow replication by others?

No

Reviewer Expertise:

Entrepreneurship and SMEs performance

I confirm that I have read this submission and believe that I have an appropriate level of expertise to confirm that it is of an acceptable scientific standard, however I have significant reservations, as outlined above.

# Response

**Published:** 2025-11-20

**Authors:** VICTOR HUGO PUICAN RODRIGUEZ

**Affiliations:** Amazonas, Universidad Nacional Intercultural Fabiola Salazar Leguía de Bagua, Bagua, Bagua, Peru

Dear reviewer,

 I sincerely appreciate the time you have taken to evaluate the manuscript and your detailed comments, which have been extremely useful in substantially improving the scientific quality and clarity of the work. Below, I present the actions taken in response to each of the points raised.

**1. Comment: The introduction is too brief.**

**Response:**

 The introduction has been completely rewritten, significantly expanded, and its theoretical foundation strengthened. The new version incorporates:

- A more in-depth discussion of the operational and financial relevance of inventory management.
- A technical explanation of replenishment policies, forecasting methods, technology adoption, and their competitive impact.
- A more solid integration with accounting literature, especially regarding IFRS/IAS 2 requirements.
- A broad contextualization of the MSME environment, with recent data and comparative analysis.
- A clear and explicit delimitation of the theoretical gaps that the study seeks to address.
- A more detailed development of the Amazonian context and the relevance of the Bagua case.

The introduction has gone from being brief to constituting a complete, coherent, and rigorous section with a solid conceptual foundation.

**2. Comment: The bibliography ignores most of the relevant recent studies. At least 30 or 40 additional articles need to be consulted.**

**Response:**

 A comprehensive set of recent literature (2022–2025) has been incorporated, sourced from journals indexed in Scopus, Web of Science, and other high-impact databases. In total, 30 new references were added, selected for their conceptual relevance to:

- inventory management,
- dynamic capabilities,
- innovation and performance,
- inventory accounting under IFRS,
- analysis of MSMEs and emerging contexts,
- impact of technologies and market turbulence.

The studies you suggested were also included, including:

- Asad et al. (2024),
- Awain et al. (2025),
- Sulaiman et al. (2024).

These references were incorporated into the introduction, conceptual framework, and discussion, strengthening the relevance and depth of the theoretical basis.

**3. Comment: “The methodology is accurate, but each step needs further explanation and bibliographic support; expand the methodology and follow the necessary tests.”**

**Response:**

 The methodology section was expanded and restructured to provide greater clarity and rigor. Notable improvements include:

- Expansion of the research design, justifying the choice of OLS analysis based on the explanatory objectives and the nature of the data.
- More complete description of the sampling process and its limitations.
- Detailed presentation of the operationalization of variables and their theoretical justification.
- Exhaustive explanation of validity and reliability: CVR, CVI, EFA, CFA, CR, AVE, and internal consistency.
- Incorporation of statistical tests prior to analysis (Shapiro–Wilk, Levene, VIF, Durbin–Watson).
- Explicit inclusion of procedural and statistical measures to mitigate common method bias.
- Clear articulation of how these tests support the results obtained.

**4. Comment: The topic is good, but each step requires explanation and support from relevant literature.**

**Response:**

 Each section of the manuscript was reviewed to ensure that the analytical processes, methodological decisions, and findings were adequately linked to recent and relevant literature. The conceptual foundation was strengthened, particularly in the following areas:

- inventory replenishment and measurement systems,
- emerging economies and MSMEs,
- dynamic capability models,
- operational-accounting integration,
- financial impact of operational capabilities,
- technological and competitive turbulence.

This allowed us to consolidate the link between theory, method, and results.

**5. Comment: “Be sure to add more studies related to innovation and profitability.”**

**Response:**

 Recent, high-impact studies in these areas were incorporated, including the three articles you recommended.

 In addition, research was added on:

- operational innovation,
- process digitization,
- supply chain innovation,
- effects of technological turbulence on MSMEs,
- combined performance indicators.

These references were integrated into both the introduction and the literature review, reinforcing the analysis of how inventory measurement acts as a dynamic capacity and how companies can translate operational practices into financial performance.

 Thank you again for your comments. The revisions made have substantially improved the scientific, methodological, and conceptual quality of the manuscript. I look forward to any additional recommendations you may consider relevant.

 Sincerely,

 Dr. Victor Puican Rodriguez

# Reviewer report: Reviewer response for version 1

**DOI:** 10.5256/f1000research.186120.r411543

**Published:** 2025-09-16

**Authors:** Juan Carlos Quiroz-Flores

**Affiliations:** 1 Carrera de Ingeniería Industrial, Universidad de Lima, Santiago de Surco, Lima, Peru

The manuscript addresses a relevant and timely problem in the field of industrial engineering and presents findings that could be of interest to both academics and practitioners. Nevertheless, there are aspects that limit its current scientific soundness. First, the presentation of the work is only partly clear. Some sections lack precise definitions and the review of current literature is limited; expanding and updating the state of the art with more recent high-impact references would help to strengthen the context and highlight the research gap.

 Regarding the study design, it is partly appropriate but not fully explained. The methodological section does not provide sufficient detail on sampling criteria, operationalization of variables, or the validation of instruments, which makes replication by others difficult. Although the statistical analysis is included, its justification and interpretation are only partly appropriate; the authors should reinforce the robustness of the chosen techniques and explain why they are suitable for the research objectives.

 An important strength of the article is the open availability of all underlying data and supplementary materials in a public repository under a CC-BY 4.0 license. This commitment to transparency significantly increases the reproducibility and value of the work, enabling replication and secondary analysis by other researchers. However, even with data availability, the methods and analytical steps should be described more systematically in the manuscript itself to ensure clarity and replicability without external consultation.

 Finally, the conclusions are only partly supported by the results. While they are aligned with the stated objectives, in some cases they are presented with greater breadth than the evidence can fully sustain. A more cautious and data-driven interpretation is recommended.

 In summary, the article makes a potentially valuable contribution but requires improvements to achieve the necessary level of scientific rigor. Specifically, the authors should: (1) strengthen and update the literature review; (2) provide a more detailed methodological description; (3) improve the justification and clarity of the statistical analysis; and (4) refine the conclusions so that they are fully supported by the presented evidence. Addressing these points would considerably enhance the impact and credibility of the study.

Is the work clearly and accurately presented and does it cite the current literature?

Partly

If applicable, is the statistical analysis and its interpretation appropriate?

Partly

Are all the source data underlying the results available to ensure full reproducibility?

Yes

Is the study design appropriate and is the work technically sound?

Partly

Are the conclusions drawn adequately supported by the results?

Partly

Are sufficient details of methods and analysis provided to allow replication by others?

No

Reviewer Expertise:

Industrial Engineering, Process Improvement, Lean Manufacturing, Supply Chain Management, Six Sigma, Digital Transformation, Industry 4.0, Machine Learning.

I confirm that I have read this submission and believe that I have an appropriate level of expertise to confirm that it is of an acceptable scientific standard, however I have significant reservations, as outlined above.

# Response

**Published:** 2025-09-16

**Authors:** VICTOR HUGO PUICAN RODRIGUEZ

**Affiliations:** Amazonas, Universidad Nacional Intercultural Fabiola Salazar Leguía de Bagua, Bagua, Bagua, Peru

Greetings, esteemed reviewer.

 Thank you very much for your comments on our work, which have enabled us to improve our research.

 All comments have been addressed.

 With gratitude,

 Dr Victor Puican

# Reviewer report: Reviewer response for version 1

**DOI:** 10.5256/f1000research.186120.r411548

**Published:** 2025-09-16

**Authors:** Vina Sari Yosephine

**Affiliations:** 1 Universitas Pembangunan Nasional Veteran Jakarta, Jakarta, Special Capital Region of Jakarta, Indonesia

General Summary

 The article, *“Innovations in Inventory Management to Improve the Profitability of Local SMEs,”* investigates the impact of inventory practices (control, valuation, record-keeping, and measurement) on the profitability of micro and small enterprises (MSEs) in Bagua, Peru. Using a quantitative, descriptive-explanatory design, the authors collected 200 valid responses from 83 MSEs via a 21-item Likert-scale questionnaire. Data were analyzed using descriptive statistics and multiple regression in SPSS v27, focusing on profitability indicators such as ROA, gross margin, and ROE.

 The results suggest that **inventory measurement** and **inventory control** significantly influence profitability, whereas valuation methods and record-keeping do not show significant effects. The authors conclude that improving measurement and control practices, along with adoption of digital systems, could enhance the financial performance of local SMEs.

 1. Clarity

 The article is generally well written and follows a standard IMRAD structure. However, the writing style is sometimes descriptive and informal, especially in the introduction and discussion (“the most important thing this paper does…”). The literature cited is up to date (2020–2023) and relevant, but the review is presented more as a *list of studies* rather than a critical synthesis. There is no clear theoretical framework or conceptual model guiding the study.

**Suggestion:**

 Revise the Introduction and Literature Review to critically synthesize prior work rather than listing references.

 Clearly identify the research gap and justify why Bagua, Peru, provides a meaningful contribution beyond confirming prior findings.

 Introduce or align with an established theoretical framework (e.g., Resource-Based View, Dynamic Capabilities, or Contingency Theory) to strengthen novelty.

 2. Design

 The quantitative survey approach is acceptable, but the use of convenience sampling weakens generalizability. The paper labels the design as “descriptive-explanatory” but does not justify why this is the most suitable design. Only four inventory practice dimensions are considered without clear rationale or theoretical justification.

**Suggestion**: Provide a stronger justification for the study design and selection of variables. Why were control, valuation, record-keeping, and measurement prioritized? Consider addressing common method bias and response bias, which are common risks in survey-based studies. Discuss limitations of convenience sampling more explicitly and propose how future research can address this.

 3. Methodology

 Some methodological details are included (e.g., pilot test, Cronbach’s alpha, validation by experts), but more transparency is needed.

 Tables are incomplete (Table 1 shows only mean values; regression results are summarized but lack full coefficients, p-values, and confidence intervals).

**Suggestion**

- Expand the Methods section with more detail on: Questionnaire design and item sources, reliability and validity results (report Cronbach’s alpha values, factor analysis results if available)., sampling logic (why 83 MSEs, how sectors were chosen).

4. Interpretation of Statistical Analysis

 The regression model is appropriate at a basic level and explains 40% of variance in profitability. However, the analysis is limited to simple regression. No robustness checks, mediation/moderation analysis, or multivariate modeling were attempted. Interpretation of results is shallow—conclusions mainly restate statistical findings without linking back to theory.

**Suggestion:**  Report full regression output, including coefficients, SE, t-values, and significance. Consider using more advanced methods (e.g., SEM-PLS or CFA) to validate the measurement model. Deepen the discussion by linking findings to prior studies and theoretical implications, not only descriptive interpretations.

 5. ConclusionThe main conclusions (measurement and control matter more than valuation and record-keeping) are supported by the data. However, broader claims about competitiveness and profitability improvement are overstated given the narrow sample and cross-sectional design.

**Suggestion**:  Revise the Conclusion to better align with study limitations. Emphasize that findings are *context-specific* (Bagua SMEs) and cannot be generalized without caution.

 Add practical managerial implication **s** for SME managers (e.g., training, adoption of digital systems).

**Minor Improvements**: Revise language for conciseness and academic tone. Improve tables and figures (add clarity, labels, descriptive statistics).Remove informal phrasing in abstract and discussion.

Is the work clearly and accurately presented and does it cite the current literature?

Partly

If applicable, is the statistical analysis and its interpretation appropriate?

Partly

Are all the source data underlying the results available to ensure full reproducibility?

Partly

Is the study design appropriate and is the work technically sound?

Partly

Are the conclusions drawn adequately supported by the results?

Yes

Are sufficient details of methods and analysis provided to allow replication by others?

Partly

Reviewer Expertise:

Industrial Engineering, Supply Chain Management, Production Scheduling, Inventory Control, Digital Transformation in SMEs, and IoT-based Logistics Systems

I confirm that I have read this submission and believe that I have an appropriate level of expertise to state that I do not consider it to be of an acceptable scientific standard, for reasons outlined above.
